# Doppler Assessment of Hepatic Venous Waves for Predicting Large Varices in Cirrhotic Patients

**DOI:** 10.4103/1319-3767.74465

**Published:** 2011

**Authors:** Thomas Joseph, Mukunda Madhavan, Krishnadas Devadas, Vinayakumar K. Ramakrishnannair

**Affiliations:** Department of Medical Gastroenterology, Medical College, Thiruvananthapuram, Kerala, India

**Keywords:** Doppler, hepatic venous waveform, varices

## Abstract

**Background/Aim:**

Color Doppler examination of changes in hepatic venous waveforms is being evaluated as a means of prediction of severity of portal hypertension and presence of esophageal varices. Normal hepatic venous waveform shows a triphasic pattern. In cirrhosis, this pattern changes to a biphasic or monophasic pattern. We aimed to study the sensitivity of loss of normal hepatic venous waveforms in predicting large varices in a cross-sectional analysis.

**Materials and Methods:**

All patients, admitted or attending the outpatient department, with a diagnosis of cirrhosis were included in the study. All patients were subjected to oesophagogastroduodenoscopy and Color Doppler examination, and waveform patterns in hepatic vein were recorded. The sensitivity and specificity of changes in waveform in detecting large varices were studied.

**Results:**

A total of 51 cases were examined. Triphasic waves were seen in 4 (7.8%) cases, biphasic in 26 (51%) cases, and monophasic in 21 (41.2%) cases. Small varices were seen in 30 (58.8%) cases and large varices in 21 (41.2%) cases. The sensitivity of loss of the triphasic wave pattern in detecting significant varices (Grade 3 or 4) was very high (95.23%) and negative predictive value was also high (75%). Severity of liver disease as indicated by Child-Pugh and MELD scores did not correlate with changes in hepatic venous waveforms.

**Conclusion:**

Loss of triphasic hepatic venous waveform is highly sensitive in predicting significant varices in patients with cirrhosis.

Acute variceal bleeding in cirrhotic patients carries a high risk of mortality. Current American Association of Study of Liver Diseases (AASLD) guidelines recommend oesophagogastroduodenoscopy (OGD) for patients with cirrhosis at diagnosis.[1] However, OGD is semi-invasive and unacceptable for some patients and also carries a small risk of complications such as perforation, aspiration and bacteremia.[2,3] Worldwide, there is a quest for non-invasive tests for detection of varices in patients with cirrhosis. Various Doppler indices have been used for assessing severity of portal hypertension in patients with cirrhosis.[4–10] Hepatic venous waveform changes are foremost among them. Normal hepatic venous waveform is triphasic. In cirrhosis this changes to biphasic and then monophasic mainly due to the loss of compliance of liver.[11–13] Our study was aimed to evaluate whether loss of the normal triphasic pattern in hepatic venous Doppler tracing would predict the presence of large esophageal varices in patients with cirrhosis.

## MATERIALS AND METHODS

The study was conducted in the Department of Medical Gastroenterology, Medical College, Thiruvananthapuram. This study was a prospective descriptive analytical study of 6 months duration from November 2007 to May 2008. All patients attending the outpatient department or getting admitted in the ward with a diagnosis of cirrhosis were enrolled in the study. The diagnosis of cirrhosis was made based on the clinical, biochemical, ultrasound, and endoscopic (presence of varices) findings, and liver biopsy was done wherever necessary. Patients with coexistent cardiac or respiratory disease, hepatocellular carcinoma, portal vein thrombosis, acute variceal hemorrhage, and those on vasodilators/propanalol, patients who have already undergone endoscopic variceal ligation (EVL) or sclerotherapy were excluded from the study. Patients in whom the hepatic venous wave pattern could not be traced due to technical reasons were also excluded from the study. After exclusion a total of 51 patients qualified to be included in the study.

OGD was done in all patients by one gastroenterologist with more than 20 years experience in doing OGD and the video was reviewed by another expert gastroenterologist, and esophageal varices were graded as small or large according to the AASLD practice guidelines.[1] Small varices were taken as those that were less than 5 mm and large varices were taken as varices more than 5 mm.

**Figure 1:**
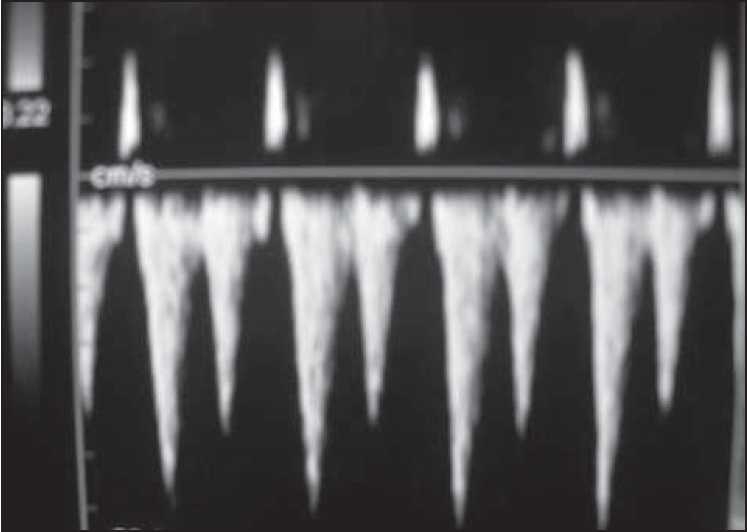
Triphasic hepatic venous wave form

**Figure 2:**
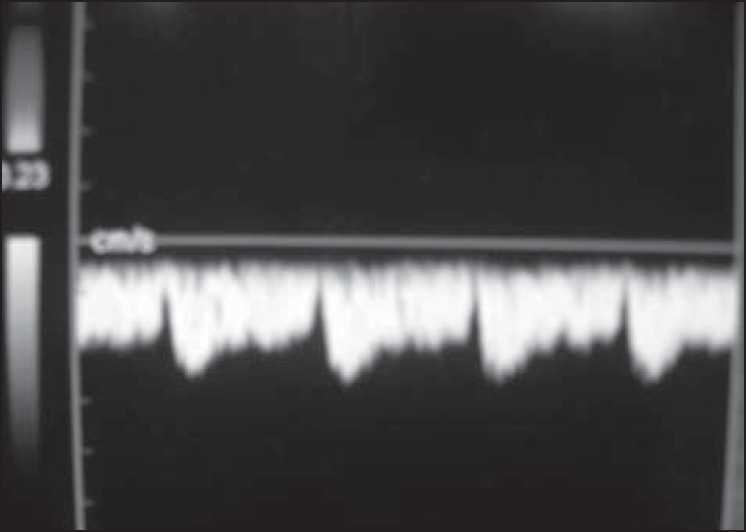
Biphasic hepatic venous waveform

**Figure 3:**
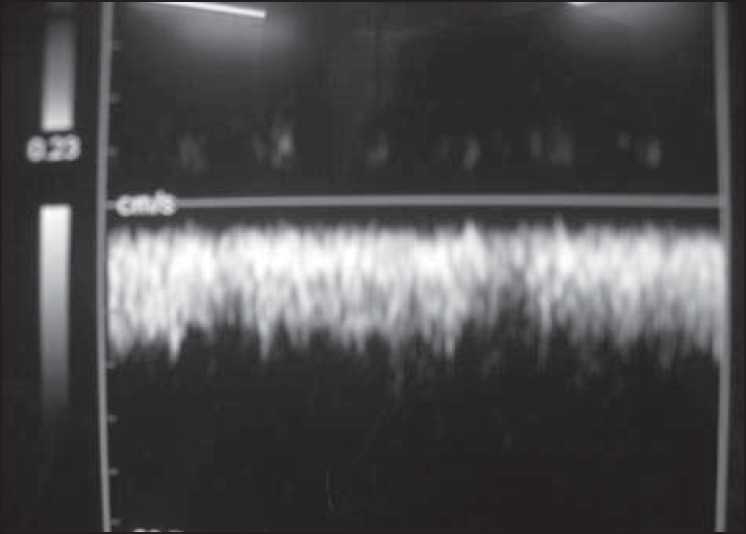
Monophasic hepatic venous wave form

All patients were subjected to ultrasound examination by two gastroenterologists who were experienced in routine ultrasound and had training in Doppler studies. They were blinded to the endoscopy findings as well as to the clinical and biochemical profile of patients. All patients underwent ultrasound examination after 8 h of fasting. First a routine B-mode examination was done and then a Doppler study was done. To trace the hepatic veins, the 3.5 MHz convex probe (Nemio Doppler Ultrasound machine from Toshiba, Japan) was placed in the right intercostal space. The hepatic veins were identified using color Doppler, and spectral analysis of the hepatic venous waveform was obtained from the right hepatic vein 3 to 6 cm from its junction with inferior venacava. When the right hepatic vein was not traced, spectral analysis was done from the middle hepatic vein. Hepatic vein Doppler waveforms were recorded for at least 5 s with end-expiration breath holding. In color Doppler flow mapping, a blue hepatic vein waveform indicates flow away from the US probe, whereas a red vein waveform indicates flow toward the US probe. We classified the hepatic vein Doppler waveform as triphasic (reversed flow in at least one phase) [Figure 1], biphasic (no reversed flow and with or without decreased phasic oscillation) [Figure 2] or monophasic (flat and with or without fluttering) [Figure 3].[11–13] Minimum of three recordings were done and if one of the recordings showed triphasic waves it was classified as triphasic, and in the absence of triphasic waves any recording showing biphasic waves was classified as biphasic, and waveform was classified as monophasic if all the three recordings were monophasic.

The size of varices was correlated with changes in hepatic venous waveforms using the Pearson chi square test. Statistical package for social sciences (*SPSS, version* 15.0; Chicago, IL, USA) was used for data analysis. The sensitivity of loss of the triphasic pattern in predicting large varices was calculated using a 2 × 2 table.

## RESULTS

A total of 51 cases of cirrhosis were examined. There were 44 men in the group and 7 women. The mean age of the group was 48.1 (Range from 24 to 78). Out of the 51 cases, 16 cases were in Child-Pugh A, 24 in Child-Pugh B, and 11 cases were in Child-Pugh C, and the mean model for end-stage liver disease (MELD) score was 7.781. 26 cases of cirrhosis were due to alcohol, 10 due to HBV, 4 due to HCV, 1 due to hemochromatosis, and 10 were cryptogenic. Hepatic venous waveform was triphasic in 4, biphasic in 26, and monophasic in 21 cases. Small varices were seen in 30 cases and large varices were seen in 21 cases. Changes in hepatic venous waveforms did not correlate with the size of the varices. However, the sensitivity of loss of the normal triphasic pattern in detecting large varices was high (95.23%). The negative predictive value was also high (75%) although the positive predictive value (42.6%) and specificity (10%) were low. Severity of liver disease as indicated by the Child-Pugh and MELD scores did not correlate with changes in hepatic venous waveforms. There was no correlation of hepatic venous waveforms with other parameters like platelet count, serum sodium, serum creatinine, serum albumin, prothrombin time, and splenic size [Table 1].

**Table 1:** Correlation of different parameters with hepatic venous waveform

Parameter	*P* value
Child-Pugh score	0.869
MELD score	0.338
Serum creatinine	0.059
Prothrombin time	0.647
Serum sodium	0.288
Splenic size	0.114
Platelet count	0.798
Serum albumin	0.217

## DISCUSSION

Variceal hemorrhage is the most common lethal complication of cirrhosis. Varices are present in around 50% of cirrhotics and their presence correlates with severity of liver disease – only 40% of cirrhotics in Child-Pugh A have varices where as it is present in 85% of cirrhotics in Child-Pugh C.[14] The strongest predictor of variceal hemorrhage is the size of the varix with the highest risk of first hemorrhage (15% per year) occurring in patient with large varices.[15–18] Upper GI endoscopy is the conventional method of detecting varices in patients with cirrhosis. However, it is a semi-invasive test and unacceptable to some patients. So Doppler ultrasound which is a non-invasive test is being tried as an alternative to endoscopy to predict the presence of varices and also to assess hepatic venous pressure gradient (HVPG).

In our study, we tried to use the Doppler parameter of hepatic venous waveforms to delineate cirrhotic patients with small and large varices. This delineation is important in the management of these patients. According to the AASLD guidelines, patients with small varices can be put on beta blockers and further endoscopic examination is not necessary in these patients unless they decompensate or bleed.[1] So we can avoid endoscopy in this group of patients if a non-invasive test like Doppler could predict the size of the varices. Furthermore, periodic Doppler examination can be used in these patients to monitor the size of varices. Doppler indexes that have been commonly used for evaluation of portal hypertension include the measurement of portal and splenic venous blood flow velocity and resistive index of splenic, hepatic, and superior mesenteric arteries. However, these indices are plagued by a lack of uniformity and accuracy due to intra and interobserver variability and interequipment variability.[19–21] However, hepatic venous waveform which is a qualitative Doppler measurement is less subject to interobserver variability and simple enough to be used clinically.

Normal hepatic venous waveform shows a triphasic pattern. Loss of this pattern in cirrhotics is mainly due to loss of compliance of liver. However, other factors also contribute to this. A recent study by Baik *et al*.[22] has shown that changes in hepatic venous waveforms are a function of HVPG and reversible with administration of Terlipressin in a small number of patients. In our study, the hepatic venous waveforms did not correlate with the size of varices. However, loss of triphasic pattern in the hepatic venous tracing had a high sensitivity in predicting the presence of large varices. The presence of a normal triphasic pattern in a patient with cirrhosis had a high negative predictive value for the presence of large esophageal varices. Earlier studies by Choi *et al*.[23] and Shabestari *et al.[24]* had shown that hepatic venous waveforms did not correlate with hepatic venous waveform changes. However, in the recent study by Baik *et al*.[22] had shown a correlation between HVPG and hepatic venous waveforms. Furthermore, Halpern in his editorial[25] has called for further studies in this direction. We feel that our study also emphasizes the role of Doppler parameter of hepatic venous waveforms in assessing portal pressure and will obviate the need of endoscopy in a group of patients with cirrhosis and portal hypertension.

Our study was limited by the fact that the sample size was small. However, this was expected because we excluded a sizable portion of our cirrhotics who presented to us with acute variceal hemorrhage as this is known to change the portal pressures. So also a considerable number of patients had to be excluded because they were on propanalol/vasodilators and some had undergone EVL/sclerotherapy. Cirrhotic patients with ascites were included in the study with the contention that ascites is not known to change the hepatic venous waveform or the size of varix although it may impair the diagnostic accuracy of the test, as hepatic decompensation along with variceal size is considered a risk factor for variceal hemorrhage.

In conclusion, the loss of the normal triphasic pattern of hepatic venous waveform is highly sensitive in predicting the presence of large varices in cirrhotic patients and this Doppler parameter may be used as a non-invasive test for cirrhotic patients who wish to avoid upper GI endoscopy. Further studies using a combination of various Doppler parameters are needed to create indices with a better predictive value.
